# The connectome‐based prediction of trust propensity in older adults: A resting‐state functional magnetic resonance imaging study

**DOI:** 10.1002/hbm.26385

**Published:** 2023-06-06

**Authors:** Yiqi Chen, Hao He, Wenyi Lin, Jiawang Yang, Siping Tan, Wuhai Tao, Qing Guan, Frank Krueger

**Affiliations:** ^1^ Center for Brain Disorders and Cognitive Sciences, School of Psychology Shenzhen University Shenzhen China; ^2^ Department of Psychology University of Mannheim Mannheim Germany; ^3^ Shenzhen‐Hong Kong Institute of Brain Science‐Shenzhen Fundamental Research Institutions Shenzhen China; ^4^ Department of Radiology Huazhong University of Science and Technology Union Shenzhen Hospital Shenzhen China; ^5^ School of Systems Biology George Mason University Fairfax Virginia USA

**Keywords:** cognitive functions, elderly, intrinsic neural connectivity, neuroeconomics, rs‐fMRI, social dilemma

## Abstract

A recent neuropsychoeconomic model of trust propensity argues that an individual uses economic (executive functions) and social (social cognition) rationality strategies to transform the risk of treachery (affect) into positive expectations of reciprocity, promoting trust in another person. Previous studies have shown that the trust of older adults is associated with affect and social cognition. However, little is known about the intrinsic functional connectivity correlated with trust propensity or whether trust propensity is associated with executive functions in older adults. In this study, we examined the association between trust propensity (measured by a one‐shot trust game [TG]), social preference (measured by a one‐shot dictator game), and executive functions (measured by a battery of neuropsychological tests). We also performed connectome‐based predictive modeling (CPM) and computational lesion analysis to identify the key large‐scale resting‐state functional connectivity (RSFC) underlying the prediction of trust propensity. Our behavioral results showed a lower trust propensity in older adults in our study than in younger adults in a previous meta‐analysis. Furthermore, trust propensity was associated with social preference, but there was no significant relationship between trust propensity and executive functions. The neuroimaging results showed that the cingulo‐opercular network (CON) and the default mode network (DMN), rather than the frontoparietal network (FPN), significantly contributed to the prediction of trust propensity in older adults. Our findings suggest that older adults rely less on economic rationality (executive functions, associated with FPN) in trust games. Rather, they are likely to depend more on social rationality (social cognition, associated with social preference and DMN) to resolve the risk of treachery (affect, associated with CON) in trust dilemmas. This study contributes to a better understanding of the neural underpinnings of older adults' trust propensity.

## Graphical abstract

We investigated the association between trust propensity, social preference, and executive functions in older adults. We used connectome‐based predictive modeling (CPM) and computational lesion analysis to identify the key large‐scale resting‐state functional connectivity underlying the prediction of trust propensity in older adults. Older adults rely less on economic rationality in trust games, and are likely to depend more on social rationality to resolve the risk of treachery in trust dilemmas.

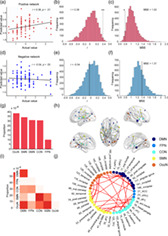

## 1 INTRODUCTION

Trust refers to the willingness of a trustor to accept vulnerability in a social dilemma based on an expectation that a trustee's action will produce an anticipated reward (Mayer et al., 1995). As an essential function of interpersonal relationships, trust is closely related to risks of fraud (Shao et al., 2019), depression (Yamaguchi et al., 2019), and well‐being (Poulin & Haase, 2015) in older adults. Trust propensity, an important component of trust, is represented by initial trust in strangers and can be assessed using a one‐shot trust game (TG) that mimics a social dilemma (Berg et al., 1995). A recent neuropsychoeconomic model of trust propensity argues that trustors promote their trust in trustees through a calculus‐based trust strategy, that is, economic (executive functions) and social (social cognition) rationality strategies, by reducing the risk of treachery (affect) in a one‐shot TG (Krueger & Meyer‐Lindenberg, 2019). Previous studies have demonstrated that the decision‐making of trust in older adults is associated with affect (Chen & Zhu, 2021) and social cognition (Chen & Wang, 2015; Fareri et al., 2022) and that they tend to rely more on social rationality when making trust decisions (Telga & Lupianez, 2021). However, little is known about the relationship between trust propensity and executive functions (decreasing with age) in older adults. In addition, prior studies have shown that trust propensity can be predicted by the large‐scale resting‐state functional connectivity (RSFC) associated with the calculus‐based trust strategy in younger adults (Bellucci et al., 2019; Feng, Zhu, et al., 2021; Lu et al., 2019). For older adults, however, the underlying RSFC of trust propensity remains to be elucidated.

Based on the neuropsychoeconomic model, trust propensity is shaped by affect, motivation, and cognition (i.e., executive, social), which engages large‐scale domain‐general networks (Krueger & Meyer‐Lindenberg, 2019). In a social dilemma created in a one‐shot TG, affect (the emotional arousal of risk of treachery) contrasts with motivation (the anticipation of reward), which produces uncertainty. Affect is anchored in the cingulo‐opercular network (CON, largely overlapping with the salience network), in which the anterior insular (AI) and anterior cingulate cortex (ACC) underlie trust propensity (Bellucci et al., 2017; Krueger & Meyer‐Lindenberg, 2019). Motivation is related to the reward network, typically involving the ventromedial prefrontal cortex, striatum, and basal ganglia (Krueger & Meyer‐Lindenberg, 2019). To resolve the uncertainty in a social dilemma, trustors can apply two types of bounded rationality, that is, economic (executive functions, adopting a context‐based strategy with extrinsic incentives) and social (social cognition, evaluating the relationship‐based trustworthiness with intrinsic incentives) rationality, to transform the risk of treachery (affect) into the positive expectation (motivation) of reciprocity (Krueger & Meyer‐Lindenberg, 2019). In a one‐shot TG, trustors reduce the uncertainty based on the calculus‐based trust strategy (i.e., trustors perform rational calculations of the costs and benefits of creating a relationship), which uses economic rationality (executive functions) to perform logical costs‐benefits calculations and social rationality (social cognition) to simulate the trustworthiness of their trustee. In particular, the CON engages the frontoparietal network (FPN, also named the central executive network) that implements a rule‐based strategy to reap personal benefits deliberately (economic rationality) and the default mode network (DMN) that evaluates the relationship‐based trustworthiness of a partner to contribute to a successful relationship (social rationality, Declerck et al., 2013; Krueger & Meyer‐Lindenberg, 2019). In addition, prior RSFC studies indicate that both the FPN (engaged in economic rationality/executive functions) and DMN (engaged in social rationality/social cognition) contribute to the prediction of trust propensity in younger adults (Feng, Zhu, et al., 2021; Lu et al., 2019).

Age‐related decision‐making studies under the dual‐process framework indicate that older adults rely more on intuitive strategies (e.g., affect and social experience) than on deliberated strategies (e.g., executive functions) in decision‐making because of the decrease in cognitive resources (Bolenz et al., 2019; Kovtun et al., 2020; Zaval et al., 2015). The engagement of the affective system in decision‐making increases with age (Finucane, 2008). Previous studies have shown that the relationship between the affective system and decision‐making performance is stronger in older adults than in younger adults (Ramchandran et al., 2020; Schiebener & Brand, 2015), indicating that the influence of affect (producing the emotional arousal of risk information) on decision‐making increases with age. The relationship between emotional arousal and trust behavior was also found to be stronger in older adults than younger adults (Filkuková & Langguth, 2021). Therefore, it is plausible that CON, which is associated with affect (Zanella et al., 2022) and risk propensity (Han et al., 2012), has an impact on trust propensity in older adults.

That older adults rely more on intuitive strategies in decision‐making suggests that they tend to use social rationality (social cognition), which depends on social experience and resolves problems in a fast and frugal manner (Hertwig & Hoffrage, 2013) to make a trust decision. Compared to executive functions, social cognition is better preserved as we age. A meta‐analysis shows that older adults perform better (close to younger adults) in solving social problems compared to instrumental and mixed problems (Thornton & Dumke, 2005). The DMN, as a core network underlying social cognition, plays an important role in older adults' socioeconomic decision‐making (Love et al., 2016). In older individuals, social cognition, including moral decision‐making (Huang et al., 2021) and theory of mind (Hughes et al., 2019), is related to the strength of the DMN. Compared to younger adults, older adults show higher DMN activation in decision‐making, which is associated with a stronger reliance on intuitive strategies (McCormick et al., 2019). Older adults show higher strength of the DMN during TG than younger adults (Fareri et al., 2022), indicating that social cognition anchoring in the DMN plays an essential role in the trust propensity of older adults.

There is much less literature on the relationship between trust propensity and executive functions in older adults. It is plausible to argue that the associations between trust propensity and executive functions decrease with age, as older adults rely less on deliberated strategies in decision‐making (Bolenz et al., 2019; Kovtun et al., 2020; Zaval et al., 2015). As such, the FPN underlying executive functions probably plays a decreasing role in decision‐making in older adults (Boggio et al., 2010; Li et al., 2017; Mell et al., 2009). Although it has been demonstrated that FPN contributes to the prediction of trust propensity in younger adults (Feng, Zhu, et al., 2021; Lu et al., 2019), the relationship between FPN and trust propensity is likely to decline in older adults, as their decision‐making relies less on executive functions.

In recent years, RSFC‐based predictions have been widely used to investigate intrinsic neural activities underlying psychological processes (Castellanos et al., 2013; Smith et al., 2013). Connectome‐based predictive modeling (CPM), a data‐driven method utilizing RSFC fMRI data (Shen et al., 2017), has been successful in predicting individual differences in cognitive abilities (e.g., attention; Rosenberg et al., 2018), social functions (e.g., decision impulse; Cai et al., 2020), and personality traits (e.g., loneliness; Feng et al., 2019). Moreover, combining computational lesion analysis with CPM is a safe and convenient method to reveal the impact of single large‐scale brain networks on the prediction of individual differences based on RSFC (Feng et al., 2018; Wang et al., 2021).

In this study, we employed a one‐shot TG and a battery of neuropsychological tests to measure the trust propensity and executive functions of older adults. We also examined the relationship between trust propensity and social preference (associated with social rationality and social cognition; Declerck et al., 2013). We used the CPM approach combined with computational lesion analysis to decode the RSFC underpinnings of trust propensity in older adults. With the hypothesis that older adults apply social rationality (social cognition, associated with DMN) rather than economic rationality (executive cognition, associated with FPN) to reduce uncertainty in the one‐shot TG, transforming the risk of treachery (affect, associated with CON) into positive expectations of reciprocity, we predicted that trust propensity would be associated with social preference, but there would be no significant associations between trust propensity and neuropsychological tests of executive functions in older adults. For RSFC, we predicted that CON and DMN, rather than FPN, would significantly contribute to the prediction of trust propensity.

## 2 METHODS

### 2.1 Participants

A total of 120 right‐handed cognitively normal older adults were recruited from communities in Shenzhen City, China. Participants had normal or corrected‐to‐normal vision and were free of head injury and neurological and psychiatric disorders. Written informed consent was obtained from all participants. The protocol was approved by the Institutional Review Board of Shenzhen University. Nineteen participants were excluded for the following reasons: (i) excessive head motion (*n* = 13, see Image Acquisition); (ii) falling asleep during scanning (*n* = 4); and (iii) disbelief of a real partner in the one‐shot TG (*n* = 2, see Procedure). A total of 101 participants (70 females, age [mean ± standard deviation] = 64.13 ± 6.39 years, education = 11.17 ± 3.21 years) were included in the behavioral and neuroimaging data analyses. The income from one of the two games (see Experimental Game Paradigms) was selected at random and used as the monetary fee for each participant. Participants received compensation as a variable monetary fee (ranging from 20 to 45 Chinese Yuan, CNY).

### 2.2 Neuropsychological tests

Executive functions are a set of cognitive processes that are necessary for goal‐directed behavior involving executive control, attention, memory, visuospatial skills, and language (Diamond, 2013; Kaushanskaya et al., 2017; Marton, 2008). We used a battery of neuropsychological tests to fully assess executive functions, including (1) executive control, assessed by the Trail Making Test Part B (Gordon, 1972), the Digital Span Test (Blackburn & Benton, 1957), and the Stroop Test (Koss et al., 1984); (2) attention, assessed by the Trail Making Test Part A (Gordon, 1972) and the Symbol Digit Modalities Test (Smith, 1973); (3) memory, assessed by the Auditory Verbal Learning Test (Schmidt, 1996) and the Rey‐Osterrieth Complex Figure Recall Test (Shin et al., 2006); (4) language, assessed by the Category Verbal Fluency Test (Mok et al., 2004) and the Boston Naming Test (Knesevich et al., 1986); and (5) visuospatial ability, assessed by the Rey‐Osterrieth Complex Figure Copy Test (Shin et al., 2006) and the Clock Drawing Test (Shulman, 2000). We only invited cognitively normal older adults to participate in the study. Older adults who showed deficits in two measures in a domain (i.e., lower than the grand mean minus 1.5 SD of the norm; Li, Ma, et al., 2013) were identified as having cognitive impairment.

### 2.3 Experimental game paradigms

#### 2.3.1 One‐shot trust game

We required participants to perform a one‐shot TG to measure their trust propensity (Berg et al., 1995), Figure 1a. Each participant was instructed to learn the rules of the game. First, both the trustor and the trustee were endowed with 10 points (1 point = 3 CNY). Then, the trustor decided how much money to send to the trustee (*X* = 0–10 points in increments of one). The money sent by the trustor was tripled (3•*X*) by the experimenter before being passed on to the trustee. Afterward, the trustee decided on how much money to send back to the trustor (*Y* = 0 to 3•X points in increments of one). At the end of the exchange, the trustor received 10 ‐ *X* + *Y* points, and the trustee received 10 + 3•*X* ‐ *Y* points. The trust propensity of each participant was determined by the number of points (*X*) sent by him‐ or herself as a trustor in the one‐shot TG.

**FIGURE 1:**
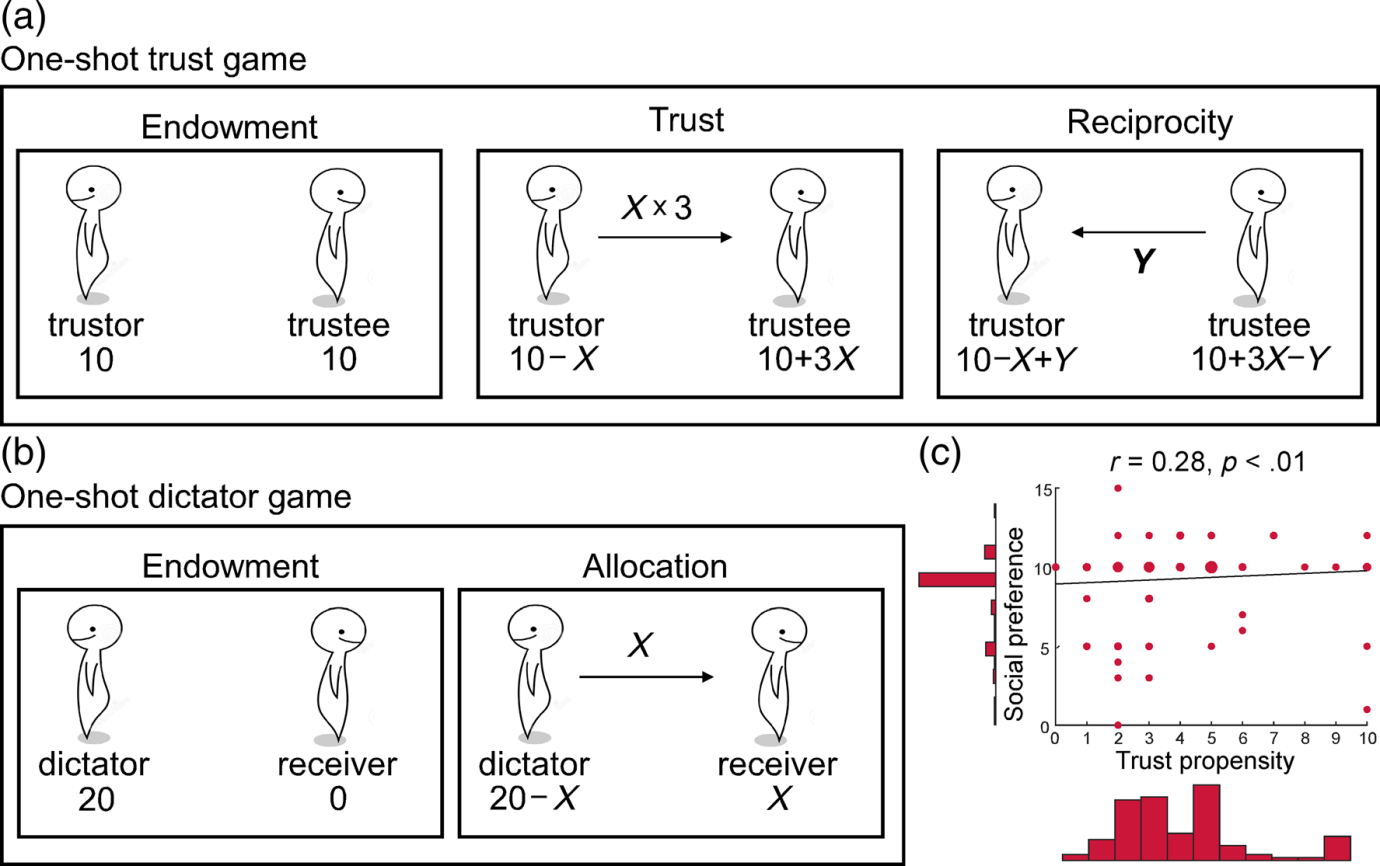
Experimental game paradigms and behavioral results. (a) The one‐shot trust game (TG). In this game, both the trustor and trustee start with 10 points (endowment). The trustor chooses to invest the trustee with an arbitrary point (Trust; *X*, 0–10), which will be tripled by the experimenter and given to the trustee. Then the trustee decides how much of the tripled investment to return to the trustor (reciprocity; *Y*, 0 to 3*X*). All participants are told that they are randomly assigned the role of the trustor and should make an investment decision (trust propensity). The returns are determined based on a computer program and range from 40% to 60% of their triple investment. (b) The one‐shot dictator game (DG). The dictator is endowed with 20 points, while the receiver is endowed with 0 points. Then, the dictator decides how many points to allocate to the receiver (*X* = 0–20). The receiver has no choice but to accept the allocation. All participants are told that they are randomly assigned the role of the dictator and determine how many points to share with the receivers (social preference). (c) Behavioral results of TG and DG. The correlation results show a significant relationship between trust propensity and social preference. A larger node size indicates a higher number of participants on that combination of trust propensity and social preference.

Participants were required to complete an exercise in which the task was to calculate the total income of the trustor and the trustee based on *X* and *Y* to ensure that they understood the task. For example, “What will be the final income for both parties if the trustor transfers 2 points (X) to the trustee and the trustee returns 4 points (Y)?” The correct answer should be that the trustor will receive 12 (10 – 2 + 4) points (36 CNY) and the trustee will receive 12 (10 + 6 – 4) points (36 CNY).

After practice, participants were informed that they were assigned the role of the trustor and that an anonymous older adult would act in the role of the trustee, who would decide how much money to return while participating in the near future in this experiment. Actually, the trustee's return was based on a random algorithm that ranged from 40% to 60% of the trustor's threefold investment, and the final payment of participants ranged from 30 to 54 CNY.

#### 2.3.2 One‐shot dictator game

An individual's social preference represents their dependency on social rationality in decision‐making, in which prosocial individuals are more likely to adopt social rationality in social dilemmas (Declerck et al., 2013). Social preference, which is measured by a dictator game (DG), is also associated with multiple social cognitions, including empathy (Edele et al., 2013) and the theory of mind (Yu et al., 2016). In this study, we used a one‐shot DG to measure participants' social preference (Forsythe et al., 1994; Figure 1b). Each participant was instructed to learn the rules of the game. First, the dictator was awarded 20 points, while the receiver was awarded 0 points. Then, the dictator decided on how many points to give to the receiver (*X* = 0–20 points in increments of one). The receiver had no choice but to accept the allocation. At the end of the allocation, the dictator received 20 ‐ *X* points, and the receiver received *X* points. The social preference of each participant was determined by the number of points (*X*) shared by him or herself as a dictator in the one‐shot DG.

Similar to the one‐shot TG procedure, participants in the one‐shot DG were required to complete an exercise in which the task was to calculate the final points of the dictator and the receiver based on *X* to ensure that they understood the rules of the game. After practice, participants were informed that they were assigned the role of the dictator and that an anonymous older adult in the near future participating in this experiment would act in the role of the receiver.

### 2.4 The assessment of emotional intelligence

Emotional intelligence, which is a reflection of the construction and regulation of emotional arousal, was evaluated using the Schutte Self‐Report Emotional Intelligence (SSREI, Schutte et al., 1998) scale. The SSREI contains 33 items and assesses four dimensions of emotion: perception (10 items), managing emotions in oneself and others (9 and 8 items, respectively), and using emotions to solve problems (6 items). Participants were asked to rate statements on the SSREI according to their own experiences using a 5‐point scale (1 = strongly disagree, 5 = strongly agree). Because the 26 participants in this study had also taken part in other experiments where their emotional intelligence was measured, we also included these data to examine the relationship between trust propensity and affect.

### 2.5 Experimental procedure

Participants performed the neuropsychological tests in communities in Shenzhen city, China, within 1 month before fMRI scanning. During the neuroimaging scanning, they completed the rs‐fMRI scan (eye‐closed, remaining awake, and without systematic thinking), lasting approximately 8 min. The participants were then required to complete a 25‐min task‐based fMRI scan (an executive function task that will be published in another study), a 7‐min high‐resolution structural scan (T1‐weight images), and a 5‐min clinical routine scan (T2‐weight images; used for medical report). After scanning, participants were instructed to complete the one‐shot DG, followed by the one‐shot TG. Finally, they were required to answer a debriefing questionnaire on their mental states and activities throughout the experiment, including questions such as “Do you believe your partner in the one‐shot TG is virtual?” and “Were you asleep during the rs‐fMRI scan?” Participants who answered yes to either question were excluded from further analysis.

### 2.6 Image acquisition

Neuroimaging data were collected with a 3T SIEMENS MAGNETOM Prisma scanner equipped with a 64‐channel head coil at Shenzhen University. High‐resolution structure brain images were acquired with a T1‐weighted 3D magnetization‐prepared rapid gradient‐echo sequence (MPRAGE) sequence (repetition time [TR] = 1.9 s, echo time [TE] = 2.23 ms, flip angle [FA] = 8°, field of view [FOV] = 220 × 220 mm^2^, voxel size = 1.1 × 1.1 × 1.1 mm^3^, 224 slices). Furthermore, rs‐fMRI brain images were acquired with a multiple band echo‐planar imaging (EPI) sequence (total volumes = 315, TR = 1.5 s, TE = 30 ms, FA = 75°, FOV = 192 × 192 mm^2^, voxel size = 2 × 2 × 2 mm^3^, 72 slices, slice thickness = 2 mm, multiband = 4, acceleration factor = 2).

### 2.7 Behavioral analysis

The statistical analyses were conducted using SPSS version 22 (IBM Corp., Armonk, NY) with a threshold of the alpha error of 0.05 (two‐tailed). The measures of trust propensity, demographic information, and neuropsychological tests were tested for normality (Kolmogorov–Smirnov test). If the assumption of normality was violated, the one‐sample Wilcoxon signed rank test, other than the one‐sample *t*‐test, was used to examine the age difference in trust propensity between our study and a previous meta‐analysis (Johnson & Mislin, 2011). Based on the detail information provided in the meta‐analysis, we computed the average transfer amount for three comparison groups: all students, all Asian adults, and all Asian students. The calculation of the average transfer amount involved multiplying the proportion of sent in each study by its corresponding number of subjects, accumulating these values, and then dividing by the total number of subjects in the comparison group. Finally, the average transfer amount of all student subjects (116 studies, 12,681 participants), all Asian adults (23 studies, 3034 participants), and all Asian students (17 studies, 1095 participants) was 51%, 48%, 58%. In addition, the Spearman correlation was used instead of the Pearson correlation to evaluate the correlation between trust propensity, neuropsychological tests, social preference, and emotional intelligence. Finally, false discovery rate (FDR) correction was used to address the multiple comparisons problem.

### 2.8 Image preprocessing

The preprocessing of neuroimaging data was performed using DPABI (Data Processing and Analysis [Resting‐State] For Brain Image, Yan et al., 2016). The preprocessing included the following steps: (1) removing the first 10 volumes to increase the stability of the signal; (2) slice timing; (3) spatial realignment; (4) grading the quality of the images and manually reorienting the images (if the origin and orientation were abnormal); (5) coregistering T1‐weight images to related mean fMRI images; (6) segmenting images with Diffeomorphic Anatomical Registration Through Exponentiated Lie algebra toolbox (DARTEL, Ashburner & Friston, 2000); (7) nuisance regression with white matter signal, cerebrospinal fluid signal, global signal, and 24 head motion parameters by the component‐based noise correction method (CompCor, Behzadi et al., 2007); (8) detrending; (9) normalizing image data to MNI space and resampling voxel size to 3 × 3 × 3 mm^3^; (10) smoothing with a Gaussian kernel (4 mm full width at half maximum); and (11) filtering (0.01–0.1). Participants with a maximum translation larger than 3 mm, maximum rotation larger than 3 degrees, or mean framewise displacement (FD) larger than 0.25 were excluded (Yan et al., 2013).

### 2.9 Construction of the RSFC matrix

The RSFC matrix was constructed based on Dosenbach's atlas, including six general large‐scale networks (160 nodes, Dosenbach et al., 2010). Because some participants' cerebellum image acquisitions were absent, the cerebellum (18 nodes) was excluded, with the remaining 142 nodes being grouped into five large‐scale networks: DMN (34 nodes), FPN (21 nodes), CON (32 nodes), sensorimotor network (SMN, 33 nodes), and occipital network (OccN, 22 nodes). For each participant, the time course of the blood oxygenation level‐dependent signal of each node was averaged from the voxels extracted from a 5 mm radius sphere centered on the node's coordinate. RSFC was measured by computing the Pearson correlation between the time courses of each pair of nodes. After Fisher's *z* transformation of the correlation coefficients, a 142 × 142 symmetric matrix was generated, representing the RSFC profile of each participant.

### 2.10 Connectome‐based predictive modeling

**FIGURE 2:**
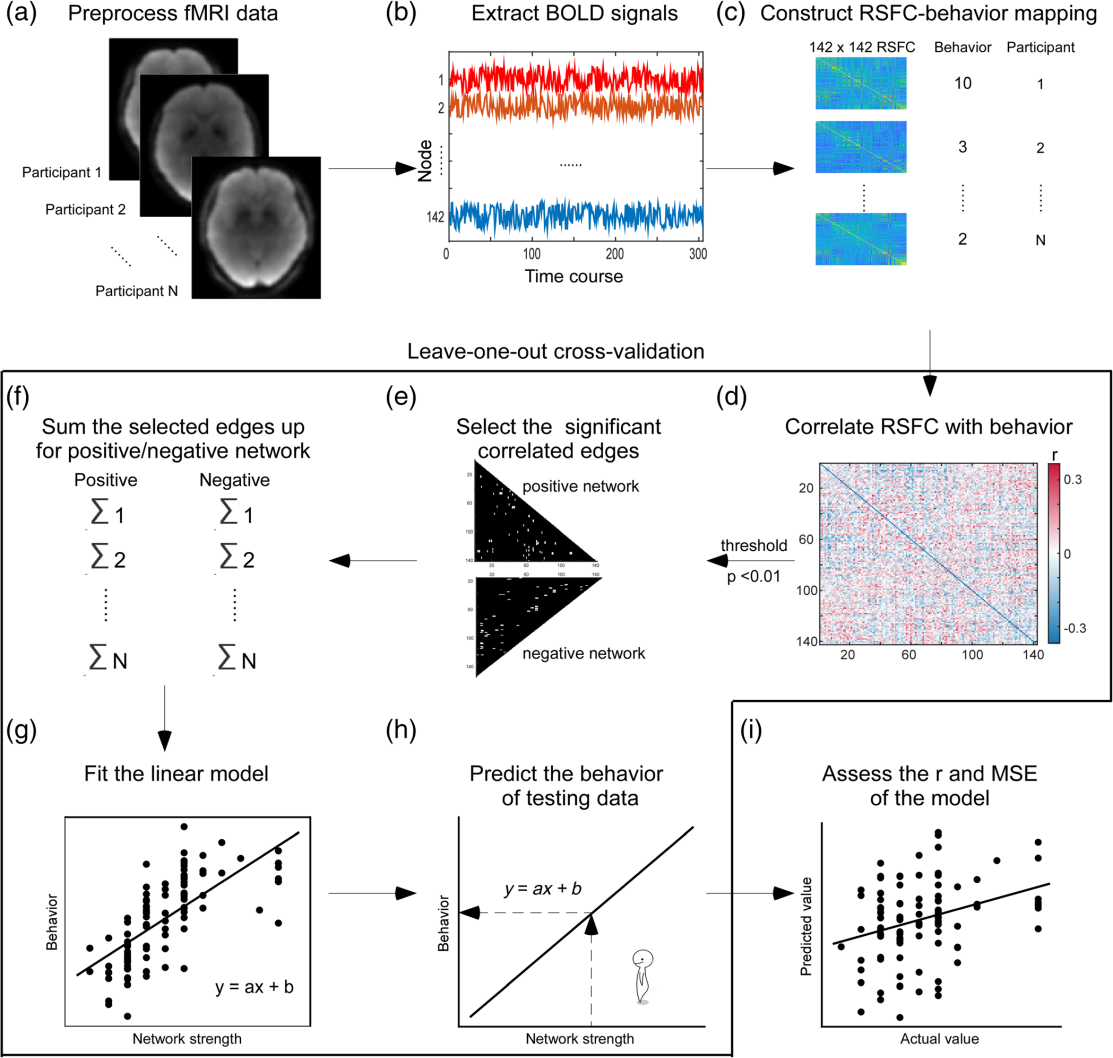
The schematic flow of connectome‐based predictive modeling. (a) Preprocessing. The resting state magnetic resonance imaging (rs‐fMRI) of each participant was preprocessed. (b) The signals extraction. For each participant, the time course of the blood‐oxygen‐level‐dependent (BOLD) signal of each node was extracted from the node's coordinate based on the Dosenbach atlas. (c) RSFC‐behavior mapping. The behavioral measure (trust propensity) and the resting‐state functional connectivity (RSFC) matrix were computed for each participant. The area in the solid‐line part represented the flow of the leave‐one‐outcross‐validation (LOOCV; each participant was used as testing data once, and other participants served as the training data). (d) The correlation matrix with RSFCs and behavior. In LOOCV, the Spearman correlation between RSFC and behavior was estimated in training data. (e) Feature selection. The edges that passed the threshold (*p* < .01) were included in the subsequent analysis and classified into positive or negative networks based on their positive/negative correlation with behavior. (f) The summation of selected edges. The network strengths of the positive and negative networks were obtained by summing up all edges of their network, respectively. (g) The fitting of linear models. The linear models between behavior and network strengths of both positive and negative networks were constructed on the training data set, respectively. (h) Prediction. The trained models were used to predict the behavior of testing data. (i) Models assessment. After the LOOCV, the Spearman correlation (*r*) and mean square error (MSE) between the predicted and actual behavior are calculated to assess the performance of the predictive model.

To predict trust propensity, CPM was applied based on the RSFC matrix (Gao et al., 2020; Shen et al., 2017). Based on the guidelines for neuroimaging‐based prediction (Poldrack et al., 2020; Scheinost et al., 2019), a leave‐one‐out cross‐validation (LOOCV) approach was suitable for a small sample size (*n* < 200). In each LOOCV iteration (Figure 2), N‐1 participants (*n* = 101) served as the training data, and the remaining participant served as the testing data. In each iteration, the training data were processed in the following steps: (1) normalization of the trust propensity and the RSFC; (2) feature selection, in which due to the nonnormal distribution of trust propensity, partial Spearman correlations (controlling the age, sex, years of education, and head motion) between trust propensity and each edge in the RSFC matrix were computed and the significant edges (*p* < .01) were selected and divided into positive (i.e., positively correlated with trust propensity) and negative (i.e., negatively correlated with trust propensity) networks; (3) network strength calculation by summing all selected edges in the positive or negative network; and (4) linear regression model training, which fit the relationship between normalized trust propensity and the strengths of the positive and negative networks.

For testing data, the trust propensity and the RSFC matrix were normalized based on the normalization parameters generated from the training set (i.e., mean and standard deviation). The selected positive and negative edges in the training dataset were chosen and summed separately to acquire the positive and negative network strength. Then, the positive and negative network strengths were separately input into the trained positive and negative models to predict trust propensity.

To illustrate the positive and negative networks, common edges were identified if they were selected in each LOOCV iteration. The common edges were divided into five large‐scale brain networks and 15 network pairs (e.g., DMN‐CON, DMN‐SMN, and CON‐SMN). In the case of between‐network common edges, they were treated as common edges in both of its two large‐scale networks while computing the number of common edges. Considering the effect of network size on the number of common edges, the proportion was used to represent the proportion of common edges in each network. Specifically, the proportion of common edges in each network was computed by dividing the number of common edges belonging to that network by the total number of edges in that network.

### 2.11 Model assessment

The Spearman correlation coefficient (*r*) between predictive and actual trust propensity and the mean square error (MSE, average difference between predictive and actual values) were computed to evaluate the performance of the predictive models. Permutation tests were performed to assess the significance of positive and negative models. In each iteration, we shuffled the true brain‐behavior mapping by randomizing the participant labels and applied the same procedure of estimating the predictive models to compute the *r* and MSE between the actual and predicted trust propensity values. The null hypothesis distributions of *r* and MSE of the positive and negative models were generated after 5000 permutations were completed. The *p* value of *r* was defined as the ratio of permutation‐generated r higher than the actual *r*, and the *p* value of MSE was defined as the ratio of permutation‐generated MSE lower than the actual MSE. The significance level of the permutation test was 0.05.

### 2.12 Lesion simulation

To understand the contribution of different large‐scale networks to predicting trust propensity, CPMs were constructed with a connectivity matrix that removed each of the five large‐scale networks of Dosenbach's atlas. For example, after removing the SMN (33 nodes), the prediction of trust propensity would be based on the remaining 109 × 109 RSFC matrix. Finally, Steiger's *z* tests (one‐tailed) were performed to examine whether the *r* (i.e., Spearman correlation coefficient between predicted and actual trust propensity values) of the lesion models was significantly lower than that of the whole‐brain model (Steiger, 1980). If *r* decreased significantly in a lesion model, the simulated lesion network served a significant role in predicting trust propensity. Finally, FDR correction was used to address the multiple comparisons problem.

### 2.13 Tenfold cross‐validation

Because of the possible overfitting problem of the LOOCV approach (Poldrack et al., 2020), a tenfold cross‐validation‐based CPM was applied as a control analysis. In the tenfold cross‐validation, the whole dataset was randomly divided into 10 folds. One served as the testing set, and the other nine folds served as the training set. We constructed the trust propensity‐associated CPMs using the procedure described earlier. We repeated the tenfold cross‐validation 100 times and used the mean r and MSE as the performance of the prediction. Permutation tests (5000 iterations) were performed to evaluate the significance of the performance (i.e., *r* and MSE) of tenfold cross‐validation.

## 3 RESULTS

### 3.1 Behavioral results

The behavioral data from participants are presented in Table 1, including trust propensity, social preference, emotional intelligence, and performance on a battery of neuropsychological tests. The average investment (i.e., trust propensity) in the one‐shot TG was 4.08 points (41% of the endowment), with an SD of 2.41 points (24% of the endowment). The distribution of trust propensity was not normal (Kolmogorov–Smirnov test statistic = 0.18, *p* < .001). The one‐sample Wilcoxon signed rank test showed that trust propensity was significantly lower in older adults in our study than all students, all Asian adults, and all Asian students in the meta‐analysis (*z* = 4.91, *p* < .001; *z* = 3.60, *p* < .001; *z* = 5.73, *p* < .001; Johnson & Mislin, 2011). The correlation results showed a significant relationship between trust propensity and social preference (*r* [100] = 0.28, *p* = .005; Figure 1c) and emotional intelligence (*r* [25] = 0.41, *p* = .04). However, there was no significant relationship between neuropsychological tests and trust propensity or social preference (Table 2).

**TABLE 1:** The trust propensity, social preference, emotional intelligence, and performance on neuropsychological tests in older adults.

Behavior data	Mean (*SD*)
Trust propensity	4.08 (2.41)
Social preference	9.26 (2.37)
Emotional intelligence	131.00 (10.50)
Rey‐recall	13.48 (6.40)
AVLT‐D	6.15 (2.41)
AVLT‐A	29.88 (8.30)
Rey‐copy	32.30 (3.84)
CDT	23.76 (5.35)
CVFT	44.29 (8.84)
BNT	23.37 (3.50)
SDMT	36.19 (11.10)
TMTA	55.13 (20.18)
TMTB	149.63 (51.01)
Stroop	79.40 (22.57)
DST	11.23 (2.11)

*Note*: Except for the Trail Making Test Part A and B and Stroop test, the higher scores of other tests represent the better performance. Except for the emotional intelligence, which only included 26 participants, all other tests included 101 participants.

Abbreviations: AVLT‐D, Auditory Verbal Learning Test (Delayed recall); AVLT‐T, Auditory Verbal Learning Test (Total recall); BNT, BOSTON NAMING TEST; CDT, Clock Drawing Test; CVFT, Category Verbal Fluency Test; DST, Digital Span Test; Rey‐copy, Rey‐Osterrieth Complex Figure Copy Test; Rey‐copy, Rey‐Osterrieth Complex Figure Recall Test; *SD*, standard deviation; SDMT, Symbol Digit Modalities Test; TMTA, Trail Making Test Part A; TMTB, Trail Making Test Part B.

**TABLE 2:** The Spearman correlations between the trust propensity and social preference and neuropsychological test scores.

Neuropsychological tests	Trust propensity	Social preference
Rey‐recall	0.11	0.07
AVLT‐D	0.03	0.17
AVLT‐A	−0.03	0.14
Rey‐copy	0.06	0.09
CDT	−0.19	0.18
CVFT	0.05	0.04
BNT	−0.04	0.05
SDMT	0.10	0.09
TMTA	−0.02	0.09
TMTB	0.13	−0.13
Stroop	0.05	−0.04
DST	0.05	0.17

*Note*: There was no significant relationship between neuropsychological tests and trust propensity or social preference.

Abbreviations: AVLT‐D, Auditory Verbal Learning Test (Delayed recall); AVLT‐T, Auditory Verbal Learning Test (Total recall); BNT, Boston Naming Test; CDT, Clock Drawing Test; CVFT, Category Verbal Fluency Test; DST, Digital Span Test; Rey‐copy, Rey‐Osterrieth Complex Figure Copy Test; Rey‐copy, Rey‐Osterrieth Complex Figure Recall Test; *SD*, standard deviation; SDMT, Symbol Digit Modalities Test; TMTA, Trail Making Test Part A; TMTB, Trail Making Test Part B.

### 3.2 CPMs associated with trust propensity

**FIGURE 3:**
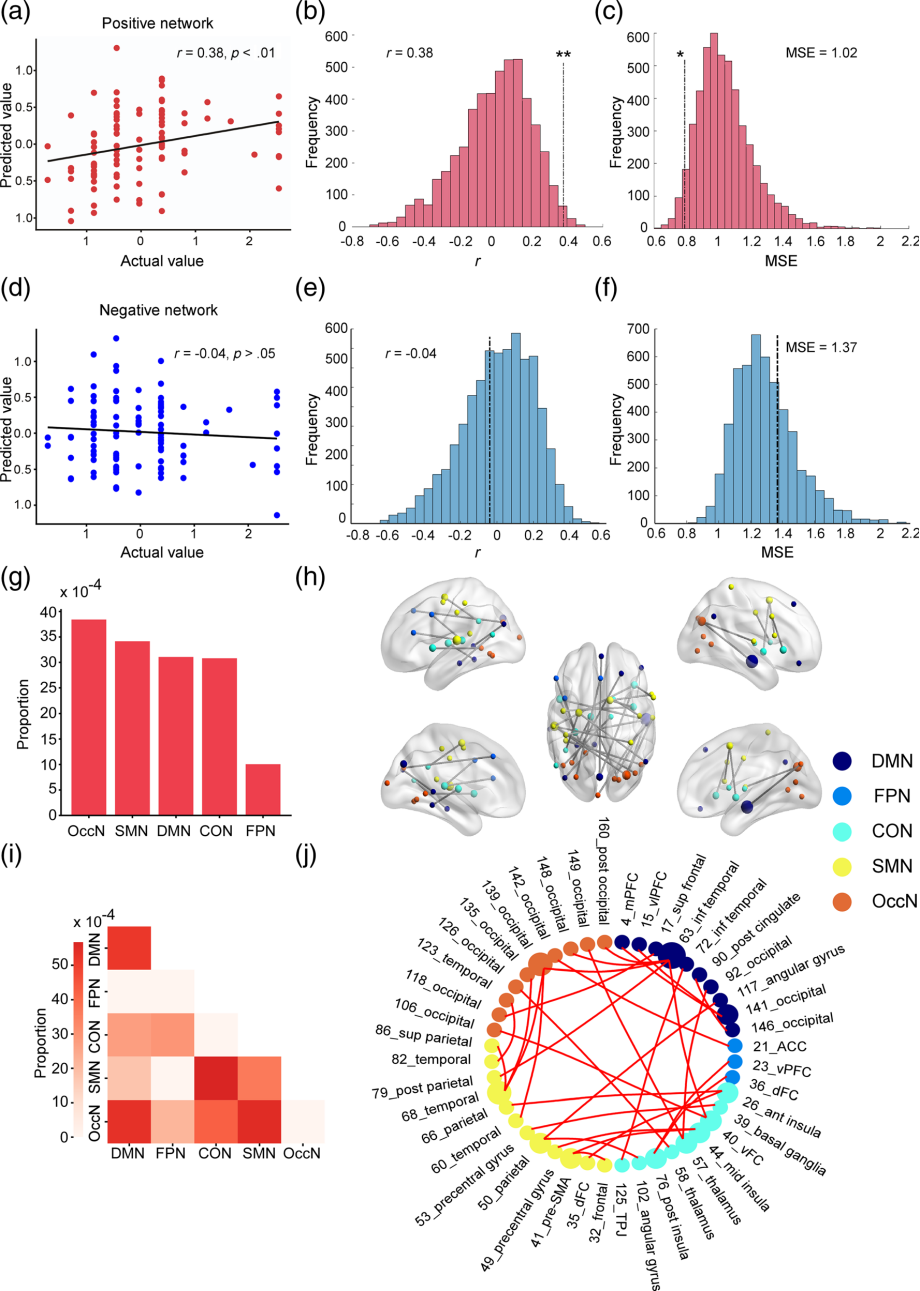
The predictive models associated with the trust propensity of older adults. (a) The prediction performance of the positive network model. The scatter plot indicates that the correlation as assessed by Spearman correlation between the actual trust propensity and the predicted trust propensity is significant in the positive network model. (b nd c) The null‐hypothesized permutation tests of the positive network model. Histograms show that both the Spearman correlation coefficient (*r*) and the mean squared error (MSE) of the positive network model are significant. The *p* value of *r* was defined as the ratio of permutation‐generated *r* higher than the actual *r*, and the *p* value of MSE was defined as the ratio of permutation‐generated MSE lower than the actual MSE. (d) The prediction performance of the negative network model. The scatter plot indicates that the correlation as assessed by Spearman correlation between the actual trust propensity and the predicted trust propensity was not significant in the negative network model. (e and f) The null‐hypothesized permutation tests of the negative network model. Histograms show that neither *r* nor MSE of the negative network model is significant. (g) The contribution of each large‐scale network in the positive network. The bar chart shows the proportion of common edges (selected in all iterations in leave‐one‐out cross‐validation) of each large‐scale network. (h) Visualization of common edges of the positive network. The brain connectome image presents the location of common edges and their involved nodes in the brain. (i) The contribution of each network pair in the positive network. The heatmap shows the proportion of common edges of each network pair in the positive network model. (j) A chord plot of the positive network. The chord plot presents the common edges of the positive network in detail. A larger size of a node in the brain connectome plot (h) or the chord plot (j) indicates that a higher number of edges are connected to that node.

The prediction performance of both positive and negative networks in predicting trust propensity was evaluated. The positive (*r* = 0.38, *p* = .04, MSE = 1.02, *p* = .05) but not the negative (*r* = −0.04, *p* = .38, MSE = 1.37, *p* = .71) model significantly predicted the trust propensity of older adults (Figure 3). To examine the positive network related to trust propensity, the proportion of common edges in each network was calculated (Figure 3g). OccN had the highest proportion of common edges. The SMN, COM, and DMN were ranked from 2 to 4. The FPN had the lowest proportion of common edges. The top 10 nodes with the most common edges were showed in Table 3. The top four network pairs with the highest proportion of common edges were CON‐SMN, SMN‐OccN, DMN‐OccN, and DMN‐DMN (Figure 3i).

**TABLE 3:** Ten nodes with the most common edges contributing to the positive prediction model.

Node	MNI coordination			Network	Degree
R_inferor temporal	52	−15	−13	DMN	5
L_temporal	−54	−22	9	SMN	3
R_Occipital	29	−73	29	OccN	3
R_anterior insula	38	21	−1	CON	2
L_vFC	−48	6	1	CON	2
R_pre‐ SMA	10	5	51	SMN	2
R_mid insula	37	−2	−3	CON	2
L_parietal	−26	−8	54	SMN	2
L_thalamus	−12	−12	6	CON	2
L_post insula	−30	−28	9	CON	2

*Note*: The node names are provided by the atlas.

Abbreviations: CON, cingulo‐opercular network; DMN, default mode network; FPN, frontoparietal network; OccN, occipital network; pre‐SMA, pre‐supplementary motor area; SMN, sensorimotor network; vFC, ventral frontal cortex.

### 3.3 Lesion simulation results

Computational lesion analyses were performed to investigate the effect of single large‐scale networks on the prediction outcomes. The exclusion of the CON or DMN significantly decreased the performance of the positive CPM in predicting trust propensity, suggesting that these two networks played key roles in the prediction of trust propensity (Table 4).

**TABLE 4:** The results of the computational lesion analysis in the positive predicted model of trust propensity.

Lesion	Predict power	Different from the whole‐brain model	
	*r*	Steiger's *Z*	*p*
DMN	0.31	2.00	0.02*
FPN	0.38	0.16	0.44
CON	0.24	2.25	0.01*
SMN	0.47	−1.73	0.96
OccN	0.38	1.18	0.12

*Note*: The *p* value is corrected by FDR correction. The *p* value with * indicates the significant results after correction (*p* < .05).

Abbreviations: CON, cingulo‐opercular network; DMN, default mode network; FPN, frontoparietal network; OccN, occipital network; SMN, sensorimotor network.

### 3.4 Tenfold cross‐validation results

To address the possible overfitting problem, CPM control analyses were performed based on tenfold cross‐validation. The results showed that the positive predictive model was significant (*r*_mean_ = 0.25, *r*_std_ = 0.08, *p* = .02; MSE_mean_ = 1.06, MSE_std_ = 0.09, *p* = .03), yet the negative model was not significant (*r*_mean_ = 0.05, *r*_std_ = 0.06, *p* = .37; MSE_mean_ = 1.30, MSE_std_ = 0.08, *p* = .57), which was consistent with the LOOCV results.

## 4 DISCUSSION

In this study, we conducted a one‐shot TG, a one‐shot DG, and a battery of neuropsychological tests to investigate whether older adults' trust propensity was associated with their social preference and executive functions, as predicted by RSFC using the CPM method. Our behavioral results demonstrated that older adults invested on average 41% of their endowment to their partners, which was lower than the investment of younger adults in a previous meta‐analysis (Johnson & Mislin, 2011). Furthermore, trust propensity was associated with social preference, but there was no significant relationship between trust propensity and a battery of neuropsychological tests. In a limited sample size, there was also a correlation between trust propensity and emotional intelligence. Our neural results revealed that the positive network, whose connectivity strength increase with the rise of investment in the trust dilemma, successfully predicted older adults' trust propensity, and the CON and DMN, rather than the FPN, significantly contributed to the prediction of trust propensity, indicating that older adults' trust propensity relies less on executive functions.

### 4.1 Trust propensity may be decreased across lifespan

According to a meta‐analysis of one‐shot TG studies, cognitively normal older adults in our study made an average investment of 41% of their endowment, which was lower than the average investment (48%) that Asian younger adults generally send to their partners (Johnson & Mislin, 2011). Out of three recent studies using a similar one‐shot TG with younger adults, two studies reported a higher trust propensity than our study, with results of approximately 50% (Bellucci et al., 2019), 42% (Lu et al., 2019), and 40% (Bayat et al., 2023). A large‐sample cross‐sectional study of one‐shot TG found that trust propensity decreases with age (Josef et al., 2016). This finding supports the findings of earlier studies that showed a decline in trust propensity after middle age (Fehr et al., 2003; Sutter & Kocher, 2007). However, several studies indicate an opposite aging pattern in which trust propensity in older adults is higher than that in younger adults (Bailey & Leon, 2019; Castle et al., 2012). One possible reason for this discrepancy is differences in measurements of trust propensity. A meta‐analysis shows that the aging pattern of trust varies across different measurements (Bailey & Leon, 2019). Many studies have assessed trust in a self‐report manner, which is vulnerable to social desirability biases that might make participants present themselves in a positive light and in a socially expected way (Krumpal, 2011). Older adults are particularly susceptible to this bias (Soubelet & Salthouse, 2011), which may lead to higher trust propensity. The trust propensity assessed by TGs is impervious to social desirability biases and provides a more accurate indicator of individuals' behavior in trust dilemmas (Lanz et al., 2022). Thus, the aging pattern of trust propensity among older adults obtained using TGs may be more accurate.

### 4.2 Association between trust propensity and social preference and executive functions in older adults

The behavioral results supported our behavioral hypothesis that there are no significant correlations between trust propensity and performance on a battery of neuropsychological tests, which is in contrast to the positive associations between trust behavior and cognitive functions (e.g., executive functions) in younger adults (Carl & Billari, 2014). Consistent with the previous findings in social rationality (Telga & Lupianez, 2021) and affect (Chen & Zhu, 2021), trust propensity in older adults was associated with social preference and emotional intelligence in this study. This evidence supports our hypothesis that older adults apply social rationality (social cognition) rather than economic rationality (executive cognition) to reduce uncertainty in the one‐shot TG, transforming the risk of treachery (affect) into positive expectations of reciprocity. Our results are predicted by age‐related decision‐making models based on dual‐process theories, which argue that older adults are more reliant on the intuitive system (e.g., social rationality) and less dependent on the deliberated system (e.g., executive functions) because of cognitive decline, resulting in a decreased correlation between general cognition and decision‐making (Y. Li, Baldassi, et al., 2013; Zaval et al., 2015). Our findings suggest that older adults are reluctant to make decisions by using economic rationality (executive functions) in trust dilemmas, which facilitates decision‐making for older adults in daily life (Zaval et al., 2015). On the flip side, it may increase the risk of fraud in older populations (Judges et al., 2017).

### 4.3 RSFC associated with trust propensity in older adults

Our results showing an insignificant role of the FPN in the prediction of trust propensity were predicted by our hypothesis of less engaging executive functions in trust dilemmas. FPN is central to rule‐based problem solving (Menon, 2011). Previous RSFC studies showed that the FPN served as one of the key networks in the prediction of trust propensity in younger adults, suggesting that they use the context‐based strategy to reap personal gains and suppress negative feelings induced by the risk of treachery (Feng, Zhu, et al., 2021; Lu et al., 2019). However, the strengths of functional and structural connectivity of the FPN decrease with age (Oschmann & Gawryluk, 2020; Zhao et al., 2015), reducing the efficiency of executive functions and requiring more mental resources to achieve task goals (Li et al., 2015). According to the principle of parsimony of brain activity, older adults, who have fewer cognitive resources than younger adults, tend to prefer intuitive strategies (e.g., affect and social cognition) to resolve problems (Hanoch et al., 2007; McCormick et al., 2019; Zaval et al., 2015). Thus, the FPN is likely to be less involved in dealing with trust dilemmas, and the decreased engagement of the FPN results in a reduction in working memory (Toepper et al., 2014; Yaple et al., 2019), inefficiency of emotion regulation (Baena et al., 2010), and difficulty responding under incongruent conditions (Prakash et al., 2009).

The CON, which played a significant role in the prediction of trust propensity in this study, largely overlaps with the salience network underlying affect in trust (Krueger & Meyer‐Lindenberg, 2019). Affect has an increased impact on older adults' decision‐making because of the decline in the modulation of the FPN (Finucane, 2008). A meta‐analysis has shown that the CON regions, including the insula that we identified as important in our study, are essential for older adults to make risk decisions (Tannou et al., 2021). This highlights the key impact of affect on decision‐making in older adults. In addition, the CON regions (e.g., insular and thalamus) are an important part of the neural mechanism of emotional intelligence (Smith et al., 2018) and emotional arousal (Colibazzi et al., 2010). The spontaneous activity of CON is positively associated with emotional intelligence (Zanella et al., 2022). A prior study indicated that the connectivity and spontaneous activity of nodes in the CON increased in older adults compared to younger adults, which was associated with a decrease in negative emotional arousal (Dolcos et al., 2014; St Jacques et al., 2010). Individuals with higher emotional intelligence, which is associated with better emotional regulation and lower negative emotional arousal, are more likely to have a higher trust propensity (Kryazh & Grankina‐Sazonova, 2018). Thus, older adults with a higher network strength of CON may be related to higher emotional intelligence and the lower negative emotional arousal associated with the risk of betrayal in trust dilemmas.

The DMN is involved in social cognition and plays a crucial role in our ability to understand other people's thoughts, feelings, and intentions (Mars et al., 2012). A meta‐analysis indicated that the DMN serves as a key network underlying the processing of social information (Feng, Eickhoff, et al., 2021). Additionally, the superior temporal region, which provided the most common edges in our study, is associated with theory of mind in meta‐analysis (Schurz et al., 2014). Beside the within‐networks of DMN, the DMN‐OccN was also the high proportion of common edges network pairs in DMN. A recent review shows that a specialized pathway for social perception is formed by the projection from the primary visual cortex, situated in the occipital network, into the temporoparietal junction/posterior superior temporal sulcus of the DMN (Pitcher & Ungerleider, 2021). In addition, the reduced connectivity of the DMN‐OccN is correlated with the clinical severity of social anxiety disorder (Ding et al., 2011). In the neuropsychoeconomic model of trust, the DMN, involved in social rationality (social cognition), reflects the evaluation of trustworthiness based on the prior experience of the anonymous partner (Krueger & Meyer‐Lindenberg, 2019). Previous studies have shown that the DMN plays an essential role in predicting trust propensity in younger adults (Bellucci et al., 2019; Feng, Zhu, et al., 2021; Lu et al., 2019). In older adults, the involvement of the DMN in economic and social decision‐making is increased compared to younger adults (Fareri et al., 2022; McCormick et al., 2019). Thus, the contribution of social rationality (social cognition associated with the DMN) to solving trust dilemmas probably increases in older people.

### 4.4 Limitations

Our study predicted the trust propensity from RSFC in older adults and revealed its intrinsic neural correlates. However, a few limitations should be mentioned. First, our study lacks a direct evaluation of social cognition and affect in trust dilemmas. Future studies should measure these functions through trustworthiness evaluation and emotional ratings of older adults in trust dilemmas, validating our CPM results. Second, we did not directly compare the trust propensity in different age groups. Future studies need to investigate variations in trust propensity with age, characterizing the aging trajectory of trust propensity and its neural underpinnings. Third, our findings lack the validation of the predictive model, and it is necessary to evaluate the test–retest reliability of the CPM‐based prediction of trust propensity in older adults (Korucuoglu et al., 2020). This needs to be addressed in the future. Despite these issues, our research found that the strategy of trust propensity in older adults was less based on economic rationality (executive functions). More significantly, it presents a potential biomarker in predicting older adults' trust propensity and identifying trust‐impaired individuals who are at high risk of financial distress and lack social support.

## 5 SUMMARY AND CONCLUSION

We examined the relationship between trust propensity and executive functions and identified the RSFC associated with trust propensity in older adults using a CPM approach. We found no significant relationship between trust propensity and executive functions. The DMN and CON rather than FPN played key roles in the prediction of trust propensity, suggesting that older adults rely less on economic rationality (executive functions) and more on social rationality (social cognition) to transform the risk of treachery (affect) into a positive expectation of reciprocity during the trust dilemma. Our findings support age‐related models based on dual‐process theories and provide an RSFC biomarker for predicting problems of trust function in older adults in future studies.

## AUTHOR CONTRIBUTIONS

Yiqi Chen, Wuhai Tao, Qing Guan, and Frank Krueger contributed to the study conception, design, and material preparation. Yiqi Chen, Hao He, Jiawang Yang, Wenyi Lin, and Siping Tan contribute to data collection. Yiqi Chen analyzed the data. Yiqi Chen, H.H., Wuhai Tao, Qing Guan, and Frank Krueger discussed the results and contributed to writing the paper. All authors commented on previous versions of the manuscript and approved the final manuscript.

## CONFLICT OF INTEREST STATEMENT

The authors declare no conflict of interest.

## Data Availability

Data and material related to this paper are available on request from the corresponding author (Qing Guan).
